# Primary psychosis and Borna disease virus infection in Lithuania: a case control study

**DOI:** 10.1186/s12888-016-1087-z

**Published:** 2016-11-03

**Authors:** Violeta Zaliunaite, Vesta Steibliene, Liv Bode, Aurelija Podlipskyte, Robertas Bunevicius, Hanns Ludwig

**Affiliations:** 1 Behavioral Medicine Institute, Lithuanian University of Health Sciences, Vyduno str. 4, Palanga, LT-00135 Lithuania; 2 Psychiatry Clinic, Lithuanian University of Health Sciences, Mickeviciaus str. 9, Kaunas, LT-44307 Lithuania; 3 Freelance Bornavirus Workgroup, Joint Senior Scientists, Beerenstr. 41, Berlin, D-14163 Germany

**Keywords:** Borna disease virus (BDV), Lithuania, Primary psychosis, Circulating immune complexes (CIC)

## Abstract

**Background:**

The hypothesis that microbial infections may be linked to mental disorders has long been addressed for Borna disease virus (BDV), but clinical and epidemiological evidence remained inconsistent due to non-conformities in detection methods. BDV circulating immune complexes (CIC) were shown to exceed the prevalence of serum antibodies alone and to comparably screen for infection in Europe (DE, CZ, IT), the Middle East (IR) and Asia (CN), still seeking general acceptance.

**Methods:**

We used CIC and antigen (Ag) tests to investigate BDV infection in Lithuania through a case-control study design comparing in-patients suffering of primary psychosis with blood donors. One hundred and six acutely psychotic in-patients with no physical illness, consecutively admitted to the regional mental hospital, and 98 blood donors from the Blood Donation Centre, Lithuania, were enrolled in the study. The severity of psychosis was assessed twice, prior and after acute antipsychotic therapy, by the Brief Psychiatric Rating Scale (BPRS). BDV-CIC and Ag markers were tested once after therapy was terminated.

**Results:**

What we found was a significantly higher prevalence of CIC, indicating a chronic BDV infection, in patients with treated primary psychosis than in blood donor controls (39.6 % vs. 22.4 %, respectively). Free BDV Ag, indicating currently active infection, did not show significant differences among study groups. Higher severity of psychosis prior to treatment was inversely correlated to the presence of BDV Ag (42.6 vs. 34.1 BPRS, respectively; *p* = 0.022).

**Conclusions:**

The study concluded significantly higher BDV infection rates in psychotic than in healthy Lithuanians, thus supporting similar global trends for other mental disorders. The study raised awareness to consider the integration of BDV infection surveillance in psychiatry research in the future.

## Background

A recent analysis of data from the Global Burden of Disease study (GBD 2010) revealed that mental and substance disorders are the fifth leading cause of DALYs (Disability-adjusted life years) and the leading cause of YLDs (years lived with disability), accounting for 7.4 % of global DALYs and 5.6 % of global YLDs [1]. The intriguing hypothesis of an infectious cause of or contribution to mental disorders has been considered for different viruses, e.g. herpes viruses and schizophrenia [2]. However, a link, whatsoever, to Borna disease virus (BDV) appeared as a particularly promising line of research since more than two decades [3, 4], Pros and Cons of which are still under debate [5]. Recently, a meta-analysis on infectious agents and depression further supported BDV as the most relevant candidate agent and found statistical significance that depressed individuals are 3.25 times more likely to be infected by BDV than healthy [6].

Borna disease virus (BDV-1; genus *Bornavirus*, species *Mammalian 1 bornavirus*) has been found worldwide [5, 7]. All variants/strains are characterized by highly conserved single-stranded, non-segmented RNA genomes (less than 10 kb) of negative polarity which replicate in the nucleus [8], persistently infecting neurons and glia cells mainly in the central nervous system. They have been shown to cause neurological diseases in a wide range of mammals, including Borna disease in horses and sheep [8, 9]. Human isolates have been reported from Germany [10, 11] and Japan [12]. Compared to genetically closely related worldwide BDV (−1) strains, recently discovered virus species in birds and reptiles were considerably different [13], including a variegated squirrel 1 bornavirus (VSBV-1) proposed to underlie three human cases of fatal viral encephalitis [14]. Independently, the finding of endogenous Borna-like N protein elements (EBLNs) which had been integrated during million years of co-evolution into germ-lines of humans and their predecessors [15–17] promoted the “Mood virus hypothesis” linking BDV with psychiatric diseases [4, 5]. After the early finding of BDV specific antibodies (Ab) in psychiatric patients [18], further worldwide evidence could also be added by the presence of BDV-specific RNA [19–29], and importantly by isolation of infectious virus either from blood cells or brain of mentally ill patients [10, 12]. However, differing sensitivity levels of antibody and RNA techniques hampered the comparability of reported infection rates. Failure of detection of any markers in psychiatric patients occurred as well despite of earlier reported evidence [22, 30], casting doubts both on techniques and impact of human BDV infection.

The discovery of virus-specific circulating immune complexes (CIC) as the most prevalent variables of BDV infection in mammals provided both a better insight into infection dynamics and a new screening instrument which allowed comparability in epidemiological studies [31]. CIC are the result of a period of virus replication, release of abundant virus proteins (N and P; antigen [Ag]) into the plasma, and subsequent generation of antibodies (Ab) in the infected host, finally leading to Ag/Ab complexes which circulate in the blood (CIC). In accord to this dynamic process, most of Ab and Ag are bound into CIC, whereas unbound Ab as well as Ag are less frequent at the same time point [31].

The determination of CIC and Ag by an ELISA technique [31] is based on two monoclonal antibodies (mAb) directed against N- and P-protein [W1, Kfu2] [32], but specificity has been questioned by those who opposed human infection [33]. However, their negative findings could be explained by an inappropriate approach in that affinity-chromatography lacked a necessary preclearance step [34], and sensitivity lacked validation through recombinant proteins spiking negative samples in a parallel approach [33]. In contrast, CIC- and Ag-ELISA results could be validated through comparative use of a different N-protein specific mAb (Bo18) [35], characterized by other researchers [36]. Specificity of the original mAbs W1 and Kfu2 were further determined by mapping their conformational epitopes on N and P proteins, respectively, and sensitivity through recombinant N-protein (1.5–3 ng/mL) [37]. Screening for human BDV-CIC could meanwhile be successfully applied in European countries, Australia, the Middle East, and China [38–42], suggesting differing prevalence in healthy carriers of countries (11 up to 37 %).

The increasing global costs of mental illness at nearly 2.5 billion USD in 2010, with a projected increase to over 6 billion USD by 2030, are associated with a huge economic burden for the society and request urgent studies in this field [43]. Except the Czech Republic [40], no information on BDV prevalence exists in East European countries with regard of these sensitive test systems. How BDV infection is determined and whether or not the most sensitive systems are applied is of paradigmatic significance, because pathogenicity is reversely associated with infection prevalence of the pathogen [37].

The aim of this study was to test Lithuanian in-patients with primary psychosis upon their admission to the mental hospital for BDV variables (CIC and Ag), and to compare the data with those of blood donors regarded as controls. Furthermore, the study aimed to evaluate whether the severity of psychiatric symptoms among in-patients correlates with the presence or absence of BDV-specific CIC and Ag. Finally, the study aimed to put the data in context to those of other countries using the same infection variables, and thereby may add to whether or not the concept of a fairly prevalent moderate pathogen could be supported.

## Methods

### Study population

At large 180 female and male in-patients, aged between 18 and 70 years, who were suffering from acute primary psychosis and consecutively admitted to the Acute Psychosis Department of the regional mental hospital in Lithuania during a 14 months period, were invited to participate in this study. The protocol of the study and subjects’ informed consent forms were approved by the Kaunas Regional Ethics Committee for Biomedical Research of the Lithuanian University of Health Sciences (2009-10-12 No. BE-2-17). In sum 156 in-patients agreed to participate and signed the informed consent form. Exclusion criteria for the study covered history of any significant or unstable medical condition, diagnosis of psychoactive drug dependence 6 month before hospital admission, and electroconvulsive therapy (ECT) 3 months before hospital admission. Pregnant women or breastfeeding mothers were excluded as well.

Therefore, only 106 in-patients (59 %) were finally included in the study, 45 men and 61 women (mean age 38.4 years; 95 % confidence interval (95 % CI) 35.8–41.0). They were evaluated as physically healthy, according to routine physical examination, medical history, routine blood and urine tests, and provided blood samples for the assessment of BDV infection markers.

The group of blood donors consisted of 98 individuals, 67 men and 31 women (mean age 31.9 years; 95 % CI 29.4–34.3). Their blood samples were procured from the Blood Donation Centre (Kaunas, Lithuania http://www.kraujodonoryste.lt/) and served as controls. The permission for using the donors’ blood samples was approved by the Kaunas Regional Ethics Committee for Biomedical Research of the Lithuanian University of Health Sciences (2010-09-30 No. P1-72/2009). All blood donors were evaluated as healthy according to an advanced blood donor examination procedure excluding infection with HIV, HBV or HCV, any severe or unstable medical conditions, any history or current mental disorders, and any somatic and psychiatric medications.

### Psychiatric evaluation

Psychiatric diagnoses were established according to the criteria of the Diagnostic and Statistical Manual of Mental Disorders, Fourth Edition, Text Revision (DSM-IV-TR) [44] using the structured Mini International Neuropsychiatric Interview [45]. Patients who met the diagnostic criteria for schizophrenia (295.3; *n* = 65), brief psychotic disorder (298.8; *n* = 20), schizoaffective disorders (295.7; *n* = 14), and schizophreniform disorders (295.4; *n* = 7) were assigned as patients with primary psychosis. Thirty three (31.1 %) out of the 106 in-patients with primary psychosis experienced the first psychotic episode during their lifetime; the other 73 patients (68.9 %) were in psychotic relapses. The duration of mental disorder prior to this psychotic episode was from 0 to 40 years; median was 2 years, interquartile range (IQR) was between 0 and 2 years. The duration of hospitalization during acute antipsychotic therapy lasted from 4 to 55 days (mean duration: 29 ± 10 days). All 106 patients received standard antipsychotic medications validated to treat acute psychosis. The vast majority of patients (*n* = 103) received additional treatment with benzodiazepines and 26 patients additional treatment with antidepressants. The severity of psychotic symptoms was assessed twice by the Brief Psychiatric Rating Scale (BPRS): (1) the next morning after hospital admission, prior to antipsychotic therapy, and (2) on the last day of hospitalization, after acute psychosis treatment was terminated. The 18 BPRS items total score rating from 0 (symptoms not present) to 6 (extremely severe symptoms) was calculated. The change in total BPRS scores indicating the efficacy of the psychosis treatment was calculated by subtracting total BPRS scores after treatment from those before treatment. All psychiatric evaluations were carried out by the same trained psychiatrist. Demographic data and clinical diagnoses were summarised in Table 1.

**Table 1:** Demographic characteristics of study populations

Factors	In-patients	Blood donors
Total number, *n*	106	98
Mean age, years (95 % CI)	38.4 (35.8–41.0)	31.9 (29.4–34.3)
Women, *n* (%)	61 (57.5)	31 (31.6)
Primary diagnosis (DSM-IV-R), *n* (%)		
Schizophrenia (295.3)	65 (61.3)	
Brief psychotic disorder (298.8)	20 (18.8)	
Schizoaffective disorder (295.7)	14 (13.2)	
Schizophreniform disorder (295.4)	7 (6.6)	
First episode, *n* (%)	33 (31.1)	
Psychotic relapses, *n* (%)	73 (68.9)	
Prior-study duration of disorder, years (median)	0–40 (2)	
Duration of hospitalization, days (SD)	29 (10)	
Standard antipsychotic treatment, *n* (%)	106 (100)	
Additional treatment with benzodiazepines, *n* (%)	103 (97.2)	
Additional treatment with antidepressants, *n* (%)	26 (24.5)	

Diagnoses according to DSM-IV-R criteria using Mini International Neuropsychiatric Interview (Ref. [44, 45])

### Serological measurements

Venous blood samples from the in-patients’ group were drawn on the last morning of hospitalisation, after termination of acute psychosis treatment. Blood samples from the control group were collected at the Blood Donation Centre (Kaunas, Lithuania). All blood samples were centrifuged and sera stored at −20 °C. The amount of BDV-specific CIC and Ag were evaluated at a certified commercial medical laboratory (Diamedis, Bielefeld, Germany), using the standardised and patented ELISA technique [31].

#### Enzyme immuno assays (EIAs)

Following the concept of maximum versatility, all EIAs including the CIC test used the same solid phase support, volume per vial (100 μl), and buffers, as well as the same initial coating steps (1 and 2), as described previously [31]. The published protocol was applied throughout as detailed below.

#### CIC assay

Step 1—polystyrene microtiter format Maxisorp Immuno Modules (Nunc, Roskilde, Denmark) were coated with 1.8 μg ml^−1^ of AffiniPure Goat Anti-Mouse IgG, FcFragment-specific (adsorbed against human, bovine, and equine serum proteins; Jackson Immuno Research, Westgrove, PA, USA), in 10 mM sodium phosphate and 250 mM sodium chloride, pH 7.6, for 1 h at 37 °C (or overnight at 4 °C). Step 2—after washing (three times in 0.9 % sodium chloride + 0.05 % Tween 20, Ultrawash Plus, Dynatech Labs, Chantilly, VA, USA), BDV p40 and p24 mouse monoclonal antibodies (moAbs) (W1, Kfu2, hybridoma supernatants IF-antibody titer 1:2000), each diluted 1:500 in PBS (pH 7.2) + 0.05 % Tween 20 (PBS-T), were incubated for 1 h at 37 °C (or overnight at 4 °C). Step 3—after washing, serum samples, diluted 1:20 and serially two-fold in PBS-T, were incubated for 1 h at 37 °C. Step 4—after washing, Alkaline Phosphatase (AP)-conjugated AffiniPure Goat Anti-Human IgG, Fc Fragment-specific (adsorbed against mouse, bovine, and equine serum proteins; Jackson Immuno Research, Westgrove, PA, USA), diluted 1:3000 in 20 mM Tris-buffered saline pH 8.0 + 0.05 % Tween 20 (TBS-T), was incubated for 1 h at 37 °C. Step 5—after washing, freshly prepared substrate *p*-nitrophenylphosphate (pNPP) (1 mg ml^−1^) in 1 M diethanolamine buffer (pH 9.8) + 0.5 mM magnesium chloride was incubated for up to 5 min at room temperature under visible control (negative and buffer control remaining colourless). Step 6—the enzymatic reaction was stopped by the addition of 50 µl of 3 M sodium hydroxide, and read at 405 nm in a Dynatech Microplate Reader MRX.

#### Antigen assay

BDV antigens p40 (N-protein) and p24 (P-protein) present in blood plasma (pAg; N/P heterodimers included) were determined by the following EIA-protocol: Steps 1 and 2—as CIC assay. Step 3—after washing, native serum samples, diluted 1:2 and serially two-fold in PBS-T, were incubated for 2 h at 37 °C. Step 4—after washing, polyclonal rabbit anti-BDV serum (IFA-titer 1:10 000), diluted 1:1000 in PBS-T, was incubated for 2 h at 37 °C. Step 5—after washing, AP-conjugated AffiniPureGoat Anti-Rabbit IgG, FcFragment-specific (adsorbed against human serum proteins; Jackson Immuno Research, Westgrove, PA, USA), diluted 1:3000 in TBS-T was incubated for 1 h at 37 °C. After washing, steps 5 and 6 followed that of the CIC assay.

#### Evaluation of assay results

Both assays made use of the same cut-off value to distinguish between positive and negative results, facilitating user friendly direct comparison of extinction values from different assays. This was possible through adapting the initial sample dilutions in that 1:20 was used for the CIC assay and 1:2 for the Ag assay, thereby considering that CIC are likely to occur at ten times higher concentrations as free antigen in host’s serum or plasma. Equal extinction values of CIC and Ag in the same sample are therefore indicative of a ten times as high CIC than Ag amount at a given time-point of infection.

Both tests were scored negative at an extinction of ≤0.1. This cut-off was determined on the basis of randomly selected negative human sera in Germany (*n* = 44) of which the mean extinction plus three standard deviations (SD) were calculated. The data for the CIC assay were as follows: mean 0.043, SD 0.019, 3xSD 0.057 resulting in a cut-off of 0.100. The data for the Ag assay were as follows: mean 0.045, SD 0.017, 3xSD 0.051 resulting in a cut-off of 0.096.

These enzyme-linked immunosorbent assays to test CIC and Ag have been independently proposed as a kind of “gold standard” for monitoring BDV infections [46]. However, a general acceptance could not be achieved so far, as already detailed above. The presence of CIC with or without antibodies indicates a chronic infection; the presence of Ag, with or without CIC at the same time, is indicative for a currently active infection [4].

### Statistical analysis

Demographic characteristics of in-patients and blood donors are presented as mean (95 % CI [confidence interval]) for continuous variables and as number (percentages) for categorical variables. For ratio comparisons between BDV infection markers, namely CIC and Ag values of patients and healthy blood donors, the chi-square test (*χ* ^2^) was applied. BPRS scores before and after treatment, delta (Δ) BPRS, duration (years) of mental disorder, and duration (days) of hospitalization were compared in the BDV- CIC positive and BDV- CIC negative as well as in the BDV- Ag positive and BDV- Ag negative groups using the Student’s *t*-test. Linear regression analyses were applied to examine whether age, gender, duration of mental disorder, treatment with antidepressants, or Borna disease virus (BDV) infection markers (CIC and/or Ag) predicted the severity of psychosis at baseline, before treatment with antipsychotics. Data were analysed using the SPSS 21.0 for Windows, and a *p*-value of < 0.05 was considered to be significant.

## Results

According to the demographic characteristics (Table 1) the study groups differed according to age and gender in that the in-patients’ group was older (F [1] = 12.825; *p* < 0.001) with higher prevalence of women (*χ* ^2^ = 3.8; df = 1; *p* <0.001). This is, however, a frequently observed mismatch in studies which used blood donors as control group [3, 4], and could rarely be circumvented [41].

Seroprevalence data of BDV infection markers among in-patients with primary psychosis and blood donor controls are presented in Table 2. We found a significantly higher prevalence of BDV-CIC in the group of treated in-patients with primary psychosis than in the healthy donors (39.6 % vs. 22.4 %, respectively, *χ* ^2^ = 7.0; df = 1; *p* = 0.008). According to gender, we found a significantly higher prevalence of BDV-CIC among women (*χ* ^2^ = 2.5; df = 1; *p* = 0.001), but not among men (*χ* ^2^ = 3.0; df = 1; *p* = 0.086). In contrast to CIC, BDV- Ag did not show significant differences among the two study groups (6.6 % Ag positives in the patients vs. 2.0 % in the controls; *χ* ^2^ = 2.5; df = 1; *p* = 0.113). BDV- Ag was found in six in-patients with psychosis relapses (four diagnosed with schizophrenia and two with schizoaffective disorder) and one patient with a first time acute psychotic episode diagnosed as a brief psychotic disorder. The age in BDV- Ag positives in-patients ranged from 20 to 41 years. Two blood donors, evaluated as BDV- Ag positives were men aged 25 and 50 years old, respectively.

**Table 2:** Seroprevalence of BDV-specific circulating immune complexes (CIC) and antigen (Ag)

	In-patients	Blood donors-controls	*p*-value	Extinction values
Total number, *n*	106	98		
CIC positive, *n* (%)	42 (39.6)	22 (22.4)	**0.008**	
Men, *n* (%)	16 (35.6)	14 (20.9)	0.086	
Women, *n* (%)	26 (42.6)	8 (25.8)	**0.001**	
CIC negative, *n* (%)	64 (60.4)	76 (77.6)	**0.027**	≤0.12
CIC weak positive, *n* (%)	15 (14.2)	12 (12.2)		>0.12–0.15
CIC positive+, *n* (%)	19 (17.9)	8 (8.2)		>0.15–0.30
CIC positive++, *n* (%)	8 (7.5)	2 (2.0)		>0.30–0.60
Ag positive, *n* (%)	7 (6.6)	2 (2.0)	0.113	
Men, *n* (%)	5 (11.1)	2 (3.3)	0.082	
Women, *n* (%)	2 (28.6)	0 (0)	0.308	
Ag negative, *n* (%)	99 (93.4)	96 (98.0)	0.255	≤0.12
Ag weak positive, *n* (%)	5 (4.7)	1 (1.0)		>0.12–0.15
Ag positive+, *n* (%)	2 (1.9)	1 (1.0)		>0.15–0.30

Extinction values read at 405 nm microplate ELISA reader

*p*-values < 0.05 indicating statistical significance between groups. Significant differences are marked in bold

**Fig. 1:**
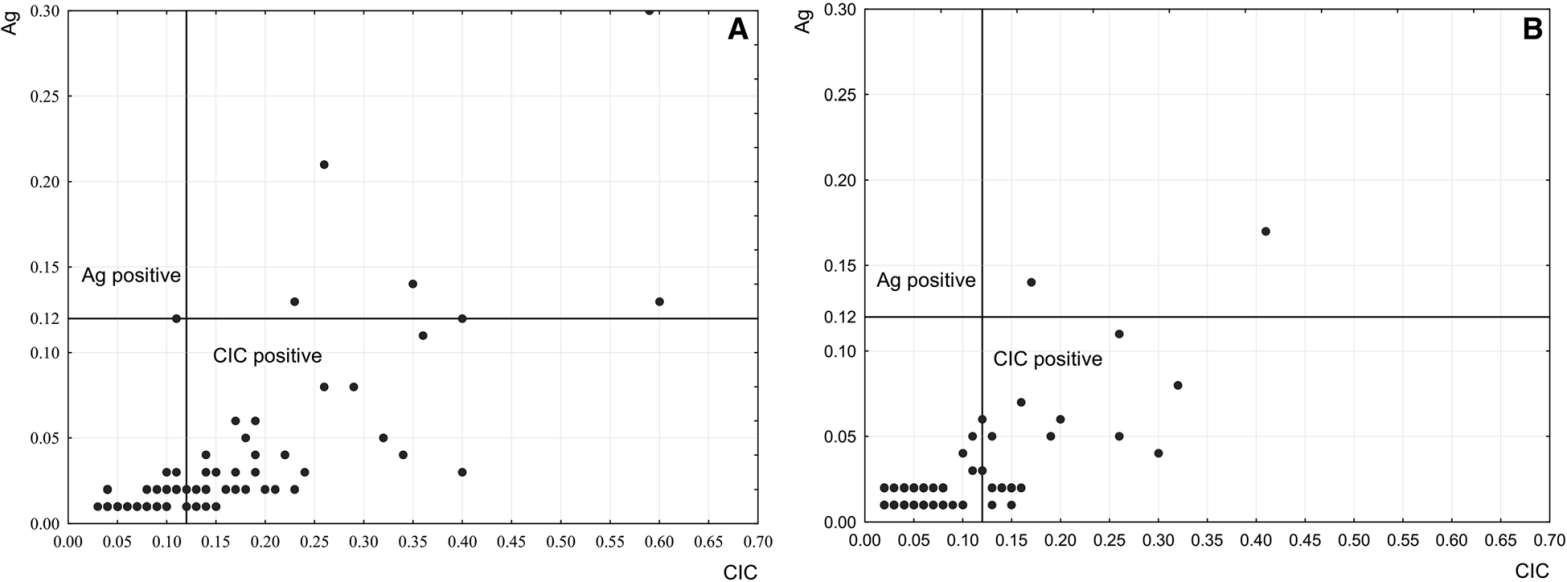
Distribution of quantitative BDV infection markers (CIC and Ag). **a** CIC-Ag pairs among patients with primary psychosis, and **b** blood donors; CIC = circulating immune complexes, Ag = antigen. Solid lines represent cut-off values of either CIC assay (*horizontal*) or Ag assay (*vertical*)

Both assays were providing quantitative measures in that extinction values corresponded to the relative amounts of CIC and Ag present at the time of serum collection, thereby considering that the relative CIC amount is ten times as high as that of Ag given different serum dilutions. CIC-Ag pairs and their distribution in patients and controls are illustrated in Fig. 1, indicating fairly similar distribution patterns between the two groups.

**Fig. 2:**
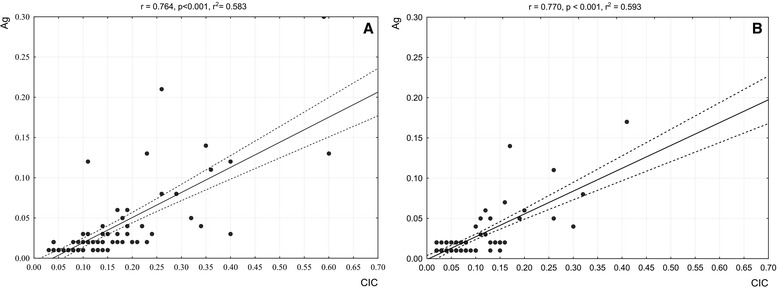
Correlation between quantitative BDV infection markers (CIC vs. Ag). Scatterplots of CIC vs. Ag extinction values determined at serum dilutions of 1:20 and 1:2, respectively; CIC and Ag abbreviations in Fig. 1. **a** data of in-patients, **b** data of blood donors; r = regression coefficient, *p* = significance level, r^2^ square regression coefficient

To further analyse to which extent CIC and Ag quantitative levels were correlated we did regression analyses of CIC vs. Ag values illustrated by scatterplots in Fig. 2. Not only could be demonstrated that CIC and Ag markers were strongly correlated to each other, but also that this correlation held true for both study groups, given the equally high correlation coefficients of *r* = 0.76 and 0.77, respectively.

We further investigated whether clinical characteristics of patients, particularly the severity of psychotic symptoms, are related to either BDV- CIC, BDV- Ag or both these markers (Table 3).

**Table 3:** Clinical characteristics and symptom severity of psychotic in-patients compared to prevalence of Borna disease virus (BDV)- CIC and -Ag

	CIC positive BDV	CIC negative BDV	p*	*p*#	Ag positive BDV	Ag negative BDV	p*	*p*#
Total number, *n*	42	64			7	99		
Mean age (95 % CI), years	35.6 (31.9–39.40)	40.2 (36.6–43.8)	0.091		29.3 (21.7–36.8)	39.1 (36.1–41.8)	0.067	
Gender			0.462				0.108	
Men, *n* (%)	16 (38.1)	29 (45.3)			5 (71.4)	40 (40.4)		
Women, *n* (%)	26 (61.9)	35 (54.7)			2 (28.6)	59 (59.6)		
BPRS before (95 % CI), score	40.6 (37.6–43.6)	42.9 (40.5–45.3)	0.225	0.375	34.1 (27.7–40.5)	42.6 (40.6–44.5)	**0.022**	**0.047**
BPRS after (95 % CI), score	21.2 (18.8–23.6)	21.2 (19.3–23.2)	0.981	0.755	16.8 (10.6–23.1)	21.6 (20.0–23.1)	0.113	0.197
ΔBPRS (95 % CI), score	19.4 (17.5–21.3)	21.7 (19.9–23.5)	0.094	0.115	17.3 (10.4–24.2)	21.0 (19.6–22.4)	0.161	0.195
Duration of mental disorder (95 % CI), years	5.2 (3.2–7.3)	6.7 (4.6–9.0)	0.356	0.882	9.0 (2.2–15.8)	5.9 (4.3–7.6)	0.337	0.050
Duration of current hospitalization (95 % CI), days	29.2 (26.0–32.3)	29.4 (27.0–31.8)	0.915	0.703	33.9 (24.7–43.0)	29.0 (27.0–30.9)	0.201	0.312

*Abbreviations*: *BPRS* Brief Psychiatric Rating Scale, *∆BPRS BPRS* score before minus, *BPRS* score after treatment, *CIC* circulating immune complexes; *Ag* antigen

p* statistical significance <0.05 significant differences are marked in bold; *p*# adjusted for age

What we surprisingly found was an inverse correlation of BDV antigen and clinical severity at baseline (prior to therapy). Using the BPRS scores, severity was higher among in-patients whose blood was negative for BDV- Ag than in those patients with a positive BDV- Ag test (42.6; 95 % CI 40.6–44.5) vs. 34.1; 95 % CI 27.7–40.5), respectively (F [1] = 5.393; *p* = 0.022). After adjustment for age, these results remained statistically significant (*p* = 0.047). In contrast, BDV-CIC were unrelated to clinical severity at baseline. Likewise, clinical efficacy of antipsychotic therapy (changes in psychosis symptoms severity; ∆BPRS) was unrelated to the presence or absence of BDV-CIC. We found no evidence for any difference between BDV-CIC positive in-patients and BDV-CIC negative in-patients with regard to either symptom severity or improvement through antipsychotic therapy.

We finally conducted a multivariate analysis to examine any relationship between psychosis severity at baseline and predictive factors (Table 4). The only significant variable for an adjusted correlation to BPRS scores prior to treatment was negatively scored BDV antigen *p* = 0.044), thereby confirming above data.

**Table 4:** Linear regression model for factors influencing severity of psychotic symptoms at baseline

Factors	Dependent variable BPRS at baseline	
	β	*p*
R^2^ = 0.127		0.041
Gender	0.078	0.443
Age, years	0.043	0.710
Antidepressants	−0.175	0.075
Duration of mental disorder, years	0.167	0.130
BDV- *CIC*	−0.069	0.502
BDV- *Ag*	−0.210	**0.044**

*Abbreviations*: *BDV- CIC* circulating immune complexes and *BDV- Ag* antigen, infection markers of BDV

*BPRS* Brief Psychiatric Rating Scale

*p* < 0.05 indicate statistical significance is marked in bold

## Discussion

This study is the first undertaking to estimate the seroprevalence of BDV (−1) infection in Lithuania by using a cross-sectional case control study design. The focus was first to investigate whether and to which levels BDV infection occurs in Lithuania, and secondly whether and how BDV infection variables CIC and Ag differs between in-patients with acute primary psychosis and healthy blood donors. The decision to determine circulating immune complexes (CIC) instead of widely used RNA and antibodies was based on higher sensitivity than achievable through the other two variables, together with high reproducibility and specificity of the enzyme immune assay (EIA) based test system [31, 37]. Comparability across countries was another profound advantage over prevailing approaches. Notably, we not only applied the virus- and host-related BDV-CIC assay as basic screening test but simultaneously determined BDV-antigen (Ag) as solely virus-derived variable in each sample.

The study not only confirmed the existence of BDV infection in Lithuania but interestingly found that the CIC prevalence in healthy Lithuanians ranged at the lowest level of 22.4 % so far reported from Europe, differing particularly from the high level of 37 % in another Eastern European country [40]. The result of one-fifth of healthy carriers did, however, support the concept that BDV is a moderate pathogen with a majority of sub-clinical infections [37].

According to patients which are a minority in populations, our study revealed a BDV prevalence of 39.6 % based on CIC which was significantly different from that of controls. Our study thus confirmed what has been reported from psychiatric patient groups in other countries (e.g. Germany, Iran, and China) using the same virus variables, all of which found a significantly higher burden of BDV infection compared to controls [4, 41, 42]. Whereas the German and Chinese studies focused solely on patients with Major depressive disorder (MDD) and bipolar depression (BD) [31, 42], the Iranian in-patients’ group additionally covered 33 patients diagnosed as having schizophrenia or schizoaffective psychosis [41]. It is noteworthy to mention that Iranian patients with schizophrenia displayed only half the CIC prevalence detected in depressed patients with mood disorders (22 % vs. 44 %) which was also much lower than in this study (39.6 %). Free BDV antigen in plasma, indicating currently active infection, reached levels of 5.6 % in Iranian schizophrenic patients, similar to our findings (6.6 %).

BDV- Ag prevalence values were too low to reach significant difference to controls in our study, and were even inversely correlated to psychotic symptoms’ severity at baseline. Possibly, the very low number of patients who were Ag-positive accounted for this somewhat tenuous finding. Moreover, it should be kept in mind that the maintenance of free antigen in plasma is largely depending on the speed and efficacy of the host to generate antibodies resulting in CIC formation [31]. The antibody EIA-test has not been available for this study. What could be clearly demonstrated, however, was the strong correlation of CIC and Ag markers which held true for both study groups, confirming the high interdependence of these variables in the course of BDV infection.

This study also evaluated whether the presence or absence of BDV-CIC determined after antipsychotic therapy correlated with severity as well as treatment variables. What we found was no correlation at all, neither in terms of severity codes by BPRS scores matching the Ag data, nor with regard to any additional treatment with benzodiazepines and/or antidepressants. We could, however, not exclude the possibility that antipsychotic treatment impact antibody levels.

The here found lack of correlation is largely contrasting previous findings covering in- and out MDD and BD patients suffering from very severe to moderate depressive episodes, whose CIC and Ag prevalence as well as marker levels were clearly corresponding to severity [4, 31]. Given comparability based on the same infection variables, this evaluation led us to speculate that BDV infection might involve more contributing links to depression (defined as MDD or BD) rather than to psychotic disorders. The Australian finding of high levels of IL-6 (>120 pg/mL) correlating with BDV antibodies in depressed patients but not in blood donors provided additional support along these lines [39]. Previous studies including schizophrenic patients, either using BDV antibody or RNA detection techniques or both, had revealed highly varying prevalence rates between 2.1 % in Poland [25], 12 % in Taiwan [24], 14 % in Germany [20], up to 22 % [26] and 45 % in Japan [23]. A more recent Chinese study found 9.9 % schizophrenic patients RNA-positive by a p24 real-time RT-PCR [29]. Lack of comparability, characteristic for many interesting studies, had not allowed to evaluate correlative evidence, whatsoever, for defined psychiatric diseases and BDV infection.

The strength of our study first lied in its high comparability with studies in other countries which were based on the determination of the same infection variables, including the most prevalent CIC. Secondly, our study investigated clinically well characterised patients with acute psychosis, and thus allowed to investigate illness- and severity-related correlations to BDV infection. Thirdly, our study included defined blood donors as healthy controls, and thus allowed to compare the percentage of silent carriers in Lithuania detected by CIC with those in other parts of the world.

The limitations of our study lied in the rather small number of subjects, the cross-sectional design, and the age mismatch of patients and control groups weakening the case-control approach. However, this kind of limitations was applying to quite a number of infection prevalence studies. The lack of general acceptance of the test systems is a clear disadvantage. However, doubts raised by an inappropriate procedural evaluation [33] appeared to be invalid as thoroughly addressed here and through previous specificity proofs [37]. Moreover, this study could clearly confirm the strong correlation of CIC and antigen in either study group adding another validity check. This may contribute to overcome current reluctance in the future.

## Conclusion

In conclusion, Lithuanians appeared to match with BDV infection patterns so far reported worldwide [31, 38–42]. The study added to comparability between countries by using highly prevalent CIC and a robust, easy-to-use and specific assay system [37] which is still competing for acceptance. One fifths of healthy Lithuanians were determined as sub-clinically infected, the so far lowest level in Europe, contrasting the significantly elevated prevalence in psychotic patients which, however, did not correlate with symptom severity. This and other case-control studies did not provide the capacity to clarify the unsettled role of BDV infection in mental disorders, but may be able to raise awareness to neglected potential risks. Given the increasing contribution of mental disorders to the global burden of disease [1] and the huge related health care costs [43], our study supports the earlier request of integrating BDV infection surveillance in psychiatry research [4]. Considering the variations in different countries, future studies should include further population variables such as the socio-economic status to address the differences that exist across countries.

## Abbreviations

Ab: Antibody
Ag: Antigen
AP: Alkaline phosphatase
BD: Bipolar disorder
BDV: Borna disease virus
BPRS: Brief Psychiatric Rating Scale
CI: Confidence interval
CIC: Circulating immune complexes
CN: China
CZ: Czech Republic
DALY: Disability-adjusted life years
DE: Germany
DSM-IV-TR: Diagnostic and Statistical Manual of Mental Disorders, Fourth Edition, Text Revision
EBLN: Endogenous Borna-like N protein
ECT: Electroconvulsive therapy
EIA: Enzyme immune assay
ELISA: Enzyme-linked immunosorbent assay
GBD: Global burden of disease
HBV: Hepatitis B virus
HCV: Hepatitis C virus
HIV: Human immunodeficiency virus
IFA: Indirect fluorescent antibody
IgG: Immunoglobulin G
IL-6: Interleukin 6
IQR: Interquartile range
IR: Iran
mAb: Monoclonal antibody
MDD: Major depressive disorder
PBS: Phosphate-buffered saline
pNPP: p-Nitrophenylphosphate
RNA: Ribonucleic acid
RT-PCR: Reverse transcription polymerase chain reaction
SD: Standard deviation
VSBV-1: Variegated squirrel 1 bornavirus
YLD: Years lived with disability

## References

[1] Whiteford HA, Ferrari AJ, Degenhardt L, Feigin V, Vos T (2015). The global burden of mental, neurological and substance use disorders: an analysis from the Global Burden of Disease Study 2010. PLoS One.

[2] Krause D, Matz J, Weidinger E, Wagner J, Wildenauer A, Obermeier M (2010). The association of infectious agents and schizophrenia. World J Biol Psychiatry.

[3] Ikuta K, Ibrahim MS, Kobayashi T, Tomonaga K (2002). Borna disease virus and infection in humans. Front Biosci.

[4] Bode L, Ludwig H (2003). Borna disease virus infection, a human mental-health risk. Clin Microbiol Rev.

[5] Ludwig H, Bode L. From latent Herpes viruses to persistent Bornavirus. In: Blaho JA, Baines JD, editors. From the hallowed halls of Herpesvirology. A tribute to Bernhard Roizman, World Scientific Publishing Co. Pte. Ltd., Hackensack, New York; 2012.169–86.

[6] Wang X, Zhang L, Lei Y, Liu X, Zhou X, Liu Y (2014). Meta-analysis of infectious agents and depression. Sci Rep.

[7] Ludwig H, Bode L (2000). Borna disease virus: new aspects on infection, disease, diagnosis and epidemiology. Rev Sci Tech.

[8] Lipkin WI, Briese T. Bornaviridae. In: Knipe D, Howley P, Griffin D, Lamb R, Martin M, Roizman B, Straus S, editors. Fields Virology. 5^th^ edition, vol. II Lippincott Williams and Wilkins, Philadelphia PA; 2007.1829–51.

[9] Ludwig H, Bode L, Gosztonyi G (1988). Borna disease: a persistent virus infection of the central nervous system. Prog Med Virol.

[10] Bode L, Dürrwald R, Rantam FA, Ferszt R, Ludwig H (1996). First isolates of infectious human Borna disease virus from patients with mood disorders. Mol Psychiatry.

[11] de la Torre JC, Bode L, Dürrwald R, Cubitt B, Ludwig H (1996). Sequence characterization of human Borna disease virus. Virus Res.

[12] Nakamura Y, Takahashi H, Shoya Y, Nakaya T, Watanabe M, Tomonaga K (2000). Isolation of Borna disease virus from human brain tissue. J Virol.

[13] Kuhn JH, Dürrwald R, Bào Y, Briese T, Carbone K, Clawson AN (2015). Taxonomic reorganization of the family Bornaviridae. Arch Virol.

[14] Hoffmann B, Tappe D, Höper D, Herden C, Boldt A, Mawrin C (2015). A Variegated Squirrel Bornavirus Associated with Fatal Human Encephalitis. N Engl J Med.

[15] Horie M, Honda T, Suzuki Y, Kobayashi Y, Daito T, Oshida T (2010). Endogenous non-retroviral RNA virus elements in mammalian genomes. Nature.

[16] Belyi VA, Levine AJ, Skalka AM (2010). Unexpected inheritance: multiple integrations of ancient bornavirus and ebolavirus/marburgvirus sequences in vertebrate genomes. PLoS Pathog.

[17] Feschotte C (2010). Virology: Bornavirus enters the genome. Nature.

[18] Rott R, Herzog S, Fleischer B, Winokur A, Amsterdam J, Dyson W (1985). Detection of serum antibodies to Borna disease virus in patients with psychiatric disorders. Science.

[19] Bode L, Zimmermann W, Ferszt R, Steinbach F, Ludwig H (1995). Borna disease virus genome transcribed and expressed in psychiatric patients. Nat Med.

[20] Sauder C, Muller A, Cubitt B, Mayer J, Steinmetz J, Trabert W (1996). Detection of Borna disease virus (BDV) antibodies and BDV RNA in psychiatric patients: evidence for high sequence conservation of human blood-derived BDV RNA. J Virol.

[21] De La Torre JC, Gonzalez-Dunia D, Cubitt B, Mallory M, Mueller-Lantzsch N, Grasser FA (1996). Detection of borna disease virus antigen and RNA in human autopsy brain samples from neuropsychiatric patients. Virology.

[22] Salvatore M, Morzunov S, Schwemmle M, Lipkin WI (1997). Borna disease virus in brains of North American and European people with schizophrenia and bipolar disorder. Bornavirus Study Group. Lancet.

[23] Iwahashi K, Watanabe M, Nakamura K, Suwaki H, Nakaya T, Nakamura Y (1997). Clinical investigation of the relationship between Borna disease virus (BDV) infection and schizophrenia in 67 patients in Japan. Acta Psychiatr Scand.

[24] Chen CH, Chiu YL, Wei FC, Koong FJ, Liu HC, Shaw CK (1999). High seroprevalence of Borna virus infection in schizophrenic patients, family members and mental health workers in Taiwan. Mol Psychiatry.

[25] Rybakowski F, Sawada T, Yamaguchi K, Rajewski A, Rybakowski J. Borna Disease Virus--reactive antibodies in Polish psychiatric patients. Med Sci Monit. 2002;8:CR642–46. 12218946

[26] Terayama H, Nishino Y, Kishi M, Ikuta K, Itoh M, Iwahashi K (2003). Detection of anti-Borna Disease Virus (BDV) antibodies from patients with schizophrenia and mood disorders in Japan. Psychiatry Res.

[27] Miranda HC, Nunes SO, Calvo ES, Suzart S, Itano EN, Watanabe MA (2006). Detection of Borna disease virus p24 RNA in peripheral blood cells from Brazilian mood and psychotic disorder patients. J Affect Disord.

[28] Li Q, Wang Z, Zhu D, Xu M, Chen X, Peng D (2009). Detection and analysis of Borna disease virus in Chinese patients with neurological disorders. Eu J Neurol.

[29] Zhang L, Xu MM, Zeng L, Liu S, Liu X, Wang X (2014). Evidence for Borna disease virus infection in neuropsychiatric patients in three western China provinces. Eur J Clin Microbiol Infect Dis.

[30] Hornig M, Briese T, Licinio J, Khabbaz RF, Altshuler LL, Potkin SG (2012). Absence of evidence for bornavirus infection in schizophrenia, bipolar disorder and major depressive disorder. Mol Psychiatry.

[31] Bode L, Reckwald P, Severus WE, Stoyloff R, Ferszt R, Dietrich DE (2001). Borna disease virus-specific circulating immune complexes, antigenemia, and free antibodies—the key marker triplet determining infection and prevailing in severe mood disorders. Mol Psychiatry.

[32] Ludwig H, Furuya K, Bode L, Klein N, Dürrwald R, Lee DS (1993). Biology and neurobiology of Borna disease viruses (BDV), defined by antibodies, neutralizability and their pathogenic potential. Arch Virol.

[33] Wolff T, Heins G, Pauli G, Burger R, Kurth R (2006). Failure to detect Borna disease virus antigen and RNA in human blood. J Clin Virol.

[34] Echan LA, Tang H-Y, Ali-Khan N, Lee K, Speicher DW (2005). Depletion of multiple high abundance proteins improves protein profiling capacities of human serum and plasma. Proteomics.

[35] Flower R, Ludwig H (2006). Presence of Borna disease virus (BDV)-specific structural components in human blood plasma. J Clin Virol.

[36] Billich C, Sauder C, Frank R, Herzog S, Bechter K, Takahashi K (2002). High-avidity human serum antibodies recognizing linear epitopes of Borna disease virus proteins. Biol Psychiatry.

[37] Bode L (2008). Human Bornavirus infection—towards a valid diagnostic system. APMIS.

[38] Patti AM, Vulcano A, Candelori E, Ludwig H, Bode L (2008). Borna disease virus infection in the population of Latium (Italy). APMIS.

[39] Flower RL, Kamhieh S, Mclean L, Bode L, Ludwig H, Ward CM (2008). Human Borna disease virus infection in Australia: serological markers of infection in multi-transfused patients. APMIS.

[40] Rackova S, Janu L, Kabickova H (2010). Borna disease virus (BDV) circulating immunocomplex positivity in addicted patients in the Czech Republic: a prospective cohort analysis. BMC Psychiatry.

[41] Mazaheri-Tehrani E, Maghsoudi N, Shams J, Soori H, Atashi H, Motamedi F (2014). Borna disease virus (BDV) infection in psychiatric patients and healthy controls in Iran. Virology J.

[42] Liu X, Bode L, Zhang L, Wang X, Liu S, Huang R (2015). Health care professionals at risk of infection with Borna disease virus—evidence from a large hospital in China (Chongqing). Virology J.

[43] Bloom DE, Cafiero ET, Jané-Llopis E, Abrahams-Gessel S, Bloom LR, Fathima S, et al. The Global Economic Burden of Noncommunicable Diseases. Geneva: World Economic Forum; 2011.

[44] DSM-IV-TR: Diagnostic and Statistical Manual of Mental Disorders. 4th ed. Text Revision edn. American Psychiatric Association, Washington; 2000.

[45] Sheehan DVL, Y Sheehan HK, Janavs J, Weiller E, Keskiner A, Schinka J (1997). The validity of the Mini International Neuropsychiatric Interview (MINI) according to the SCID-P and its reliability. Eur Psychiatry.

[46] Thakur R, Sarma S, Sharma B (2009). Role of Borna disease virus in neuropsychiatric illnesses: are we inching closer?. Indian J Med Microbiol.

